# Supplementary material for: Moms in motion: Predicting healthcare utilization patterns among mothers in Newfoundland and Labrador
Source: PLoS One. 2024 Jul 9;19(7):e0304815. doi: 10.1371/journal.pone.0304815 (PMC11233017; doi:10.1371/journal.pone.0304815)
Supplement: Attachment — Submitted filename: MomsInMotion_RevisionLetter-FINAL.docx [file pone.0304815.s001.docx]

Dear Editorial Board, PLOS One,

Thank you for considering our manuscript entitled “Moms in motion: Predicting Healthcare Utilization Patterns among Mothers in Newfoundland and Labrador” for publication in *PLOS One*. We greatly appreciate the timely and thoughtful feedback provided by both reviewers who agreed to review our work. We have taken each suggestion into consideration and provided a point-by-point response below. All corresponding changes in the manuscript have been made using track changes. We believe the feedback we have received has improved our manuscript.

Thank you and we look forward to hearing back from you.

Sincerely,

The Authors

**Reviewer 1**

1. Great article but there are few concerns. In the introduction kindly mention the current health care utilization data at least within 5 yrs till date.

**Author Response:** We appreciate the reviewers kind words about our article. We have incorporated additional citations published since 2018 to our revised manuscript.

2. How was the questionnaire validated, please state it clearly. In addition, kindly explain clearly the line 220-224 supporting and justifying the same.

**Author Response:** The survey utilized for this study was developed to create a comprehensive profile of mothers in NL that allowed for a better understanding of the population and the identification of predictors of motherhood satisfaction and maternal self-efficacy. While no statical analyses or pilot testing was completed to validate the survey, items selected were informed by review of relevant literature examining maternal and family experiences. Specifically, the survey was modelled after a Canadian survey examining maternal experiences in stepfamilies, entitled ‘Canadian Survey on Parenthood’ and from an Australian longitudinal study, entitled ‘Growing Up in Australia’, which examined the nature and impacts of early family composition, relationships, and dynamics on outcomes of interest. Minor adjustments were made to language to reflect the context within NL (e.g., for clarity around health services offered within the province). It is also important to note that the survey was composed primarily of questionnaires with strong psychometric properties, including evidence to support reliability and validity. We have clarified this in our revised manuscript, refer to lines 223-227.

3. Kindly check out the data for this study for speedy usage. It might be outdated for publication usage. Since it has to be used within 5yrs after data collection.

**Author Response:** We respectfully disagree with the notion of placing an arbitrary cut-point at which data is considered outdated for use in publication (i.e., presuming that data that is more than 5-years old is outdated for use in publication). The knowledge to practice gap in medicine is approximately 17-years which suggests that most knowledge would not make it into practice within such a timeframe (Morris et al., 2011). Further, data collection in large clinical trials can often take longer than this cut-point. We advocate for a nuanced approach, and contend that our submitted manuscript adds great value to the existing literature as the largest (and one of the only) studies to date that evaluates health care utilization superficially among mothers, refer to lines 814-821 for additional information on the dearth of literature pertaining to this topic.

Morris ZS, Wooding S, Grant J. The answer is 17 years, what is the question: understanding time lags in translational research. J R Soc Med. 2011;104(12):510–20.

**Reviewer 2**

1. Citations in this manuscript starts from number 3. Authors should begin to cite with number 1.

**Author Response:** We thank the reviewer for calling attention to this oversight on our part. We have ensured that citations begin at 1 in our revised submission.

2. Please describe introduction section using paragraphs and no need for sub headings in this section (Page 3 line 64 and Page 8 line 172).

**Author Response:** We have removed the subheadings in the introduction.

3. There are 67 citations in introduction section out of 113 references. Authors just have only 50% citation in discussion section compared to introduction. Please more comprehensive literature review in discussion section.

**Author Response:** We thank the reviewer for the opportunity to elaborate on the discussion section of our manuscript. We have taken this opportunity to strategically elaborate on results pertaining to utilization of services for mothers (refer to lines 691-705), and the effect of rurality on mothers’ access to services for their children (refer to lines 728-739).

4. The literature review is promising but disregards recent publications in the field of healthcare utilization. Please more recent literature review (From 113 referenced papers in this study, 74 references are older than 2013).

**Author Response:** Please refer to our response to Reviewer #1, Concern #1.

5. Please clearly explain in method section regarding independent and dependent variables. Information given in the method is a bit confusing for readers.

**Author Response:** We have elected to retain language in the statistical analysis section which are commonly used within regression, and have clarified which variables served as dependent variables, independent variables, and covariates for transparency. Please refer to the statistical analysis section of the revised manuscript. We also revised the manuscript to ensure consistency in referring to “STEPs” in regression models rather than “blocks.”

6. I had difficulties understanding the table 1 and table 3 and suggest that the authors simplify it. Please summarize it. If it possible, please re-categorized each variables.

**Author Response:** We appreciate that Tables 1 & 3 were lengthy and could be confusing for some readers, and have taken a number of steps to more effectively present this information. We have removed demographic categories that were not selected by participants (e.g., cohabiting but widowed category under marital status) from Table 1. We also moved the self-report data concerning produce consumption, exercise, sleep quality, health and social support to Table 2 and now only report the means and standard deviations for these variables. The title of Table 2 has now been revised to reflect these changes, refer to **Page 51, Line 1148**). Finally, we removed variables concerning satisfaction of service availability from Table 3 and provided their means and standard deviations in the written text (**Page 24, Line 527-531**). We hope that these revisions make the material presented easier to understand.
